# Flexible Coupling in Joint Inversions: A Bayesian Structure Decoupling Algorithm

**DOI:** 10.1029/2018JB016079

**Published:** 2018-10-13

**Authors:** Nicola Piana Agostinetti, Thomas Bodin

**Affiliations:** ^1^ Department of Geodynamics and Sedimentology Universitat Wien Wien Austria; ^2^ Univ Lyon, UniversitÃl' de Lyon 1, ENS de Lyon, CNRS, UMR 5276, LGL‐TPE Villeurbanne France

**Keywords:** inverse problems, joint inversions, Bayesian inferences, trans‐dimensional algorithms

## Abstract

When different geophysical observables are sensitive to the same volume, it is possible to invert them simultaneously to jointly constrain different physical properties. The question addressed in this study is to determine which structures (e.g., interfaces) are common to different properties and which ones are separated. We present an algorithm for resolving the level of spatial coupling between physical properties and to enable both common and separate structures in the same model. The new approach, called structure decoupling (SD) algorithm, is based on a Bayesian trans‐dimensional adaptive parameterization, where models can display the full spectra of spatial coupling between physical properties, from fully coupled models, that is, where identical model geometries are imposed across all inverted properties, to completely decoupled models, where an independent parameterization is used for each property. We apply the algorithm to three 1‐D geophysical inverse problems, using both synthetic and field data. For the synthetic cases, we compare the SD algorithm to standard Markov chain Monte Carlo and reversible‐jump Markov chain Monte Carlo approaches that use either fully coupled or fully decoupled parameterizations. In case of coupled structures, the SD algorithm does not behave differently from methods that assume common interfaces. In case of decoupled structures, the SD approach is demonstrated to correctly retrieve the portion of profiles where the physical properties do not share the same structure. The application of the new algorithm to field data demonstrates its ability to decouple structures where a common stratification is not supported by the data.

## Key Points

- The SD algorithm outperforms classical algorithms in recognizing volumes where two different properties are not sharing the same structure
- In case of fully coupled structures, our algorithm produces comparable solutions to those offered by standard approaches
- The algorithm has been tested on both a standard changepoints inverse problem and a more complex geophysical joint inversion

## 1 Introduction

Geophysical measurements made at the Earth's surface (e.g., gravity) can generally be related to the spatial variations of one physical property (e.g., density) within a given volume. When two or more observables are sensitive to the same target volume, one can be tempted to simultaneously invert them to constrain multiple physical properties within the volume (the so called *joint inversion*; Moorkamp, 2017). The class of procedures called joint inversions is wide (e.g., using two observables for constraining a single property is also termed joint inversion; see Kalscheuer et al., 2017). Obviously, the joint inversion of complementary observables increases the ability to resolve details of the structure in different portions of the target volume.

**Figure 1:**
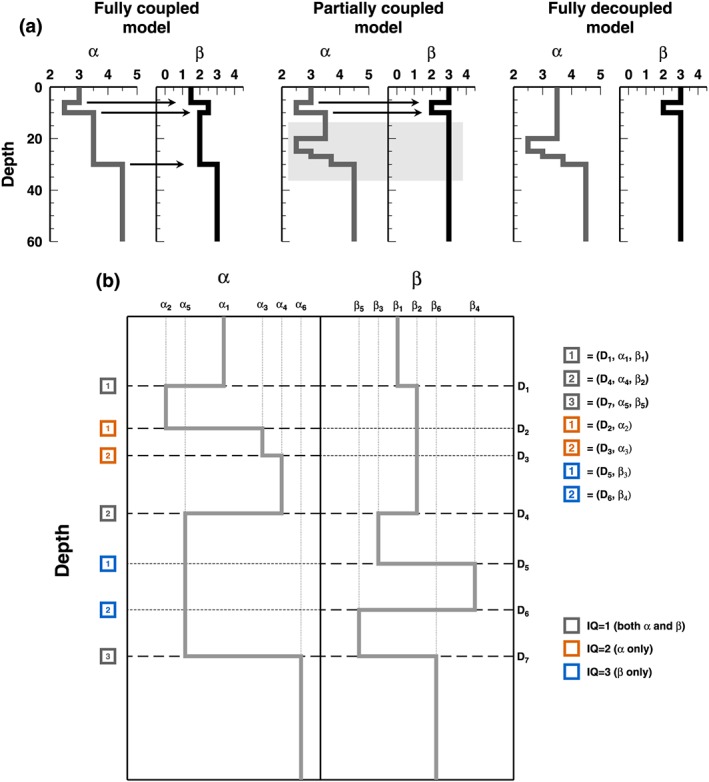
(a) Example of three 1‐D profiles for properties α and β: (left) fully coupled at depth, that is, the two profiles display the same number of interfaces, positioned at the same depths; (center) partially coupled at depth, that is, the two 1‐D profiles share two common interfaces at shallow depth, but the 1‐D profile for α presents three additional interfaces, at the depth level indicated by a gray‐shaded area; and (right) fully decoupled profiles, that is, the two 1‐D profiles display a different number of interfaces at not‐coincident depth positions. (b) A structure decoupling model for properties α and β. The model is composed of seven interfaces: k_1_=3, k_2_=2, and k_3_=2. In these examples, we are using “Depth” along one axis (here y axis), illustrating spatial variation. The same examples can be done using “Time” along x axis, to present temporal variations.

A common parameterization is often used to describe the spatial variations of the different properties. For example, one may assume that different properties share common interfaces, in which case properties are spatially correlated or coupled (Figure 1a). While a coupled parameterization can be voluntarily exploited in joint inversions, this assumption needs to be carefully investigated when the level of resolution for two properties is very different. In this situation, the structure belonging to a weakly resolved physical property may reflect the structure of a better resolved property, thus resulting in spurious structure not required by the data. In such cases, two different structures with two levels of spatial resolution should be considered. Here we term two uncorrelated structures as *decoupled* (Figure 1a). For example, two separated single inversions can be carried out independently and with different parameterizations, before joint interpretation of the results (e.g., Bedrosian et al., 2007). This fully decoupled approach is routinely done when seismic *P* wave and *S* wave travel times are separately inverted for *P* and *S* wave velocities (Caló et al., 2012). See also Evans et al. (1994) for a discussion where *V*_*P*_ velocity and *V*_*P*_/*V*_*S*_ ratio are jointly inverted for in seismic tomography.

Joint inversion approaches that account for the level of spatial coupling between two or more properties have been proposed in the past years like cross‐gradients constraints (e.g., Gallardo & Meju, 2004), adaptive coupling strategy (e.g., Heincke et al., 2017), or level‐set‐based structural parameterization (e.g., Li & Qian, 2016). In these studies, the level coupling is directly imposed by the user through a penalty term in the objective function, in the first case, or in an iterative redefinition of the coupling constraints, in the latter cases. Subjective choices are introduced in terms of weighting the relative contribution of different terms in objective function (Gallardo & Meju, 2004; Moorkamp, 2017) or selecting the number of set‐level perturbations to compute for the updating (Li & Qian, 2016). As a consequence, even if the *fully coupled structure* assumption is removed, the degree of coupling is still in the hands of the practitioner.

In Gallardo and Meju (2004, 2007), the authors penalize models for which the spatial variations (gradient) of the two properties over a 2‐D map or 3‐D volume are different, following the assumption that the same geological structure must be seen by the two observables. In the cross‐gradient approach, as in many others, a condition is introduced in the algorithm to minimize a penalty term over the entire volume (i.e., to favor “coupled” models over “decoupled” ones) leading to models with remarkably coupled structures. In this case, the level of coupling imposed by the user is global, and difference in the levels of spatial resolution is not accounted for (see, e.g., Doetsch et al., 2010).

Recently, a new algorithm has been introduced for joint inversion of different seismological observables to constrain a 1‐D seismic profile at depth (Bodin et al., 2016). The two physical properties to be investigated are the isotropic seismic velocity and the level of seismic anisotropy. The algorithm is developed following a trans‐dimensional (trans‐D) approach where the number of investigated parameters (e.g., number of layers and number of physical properties) is not fixed, but it is an unknown itself (Malinverno, 2002; Sambridge et al., 2006). Given its natural parsimony, such an algorithm guarantees that anisotropy is added to a layer as an extra parameter only if the data support it. In this way, some layers are isotropic and described by one parameter, and some layers are anisotropic and described by two parameters. This algorithm does not force the coupling of the structure (i.e., anisotropy is not inverted for in every layer and does not share the same discontinuities as isotropic velocity). While this algorithm allows to investigate partially decoupled structures, it cannot be easily generalized, due to the implicit link between the two physical properties investigated (isotropy and anisotropy).

In this study, we propose a more general algorithm for investigating the degree of spatial decoupling between physical parameters. The proposed algorithm applies to the case where two physical properties are constrained from an arbitrary number of observables, and we give some indications on how to generalize it to the case of a larger number of physical properties. We illustrate the approach using two 1‐D synthetic test cases, where two observables are inverted to constrain two physical properties. We also show a real data application using two different borehole logs, reversed delay time (DTR) and electromagnetic measurements, and comparing coupled and decoupled structures to the borehole lithostratigraphy.

### 1.1 Bayesian Inference

The term *Bayesian inferences* collectively indicates all those procedures that make some use of Bayes's (1763) theorem, where a quantitative relation is introduced for linking previous, already established, knowledge to new observations. The results are given in terms of probability for a given investigated parameter to have a certain value (i.e., a probability distribution over the physically consistent values of a parameter). Called **m** a vector of investigated parameters (i.e., a model), this relation can be expressed as

(1) $$p(m|d)=\frac{p(m)p(d|m)}{p(d)},$$

where *p*() is a probability distribution, *A*∣*B* indicates conditional dependence (i.e., *A* given *B*), and **d** denotes the observed data. Thus, *p*(**m**∣**d**) is called posterior probability distribution (PPD) and is our target distribution, *p*(**m**) is called prior probability distribution (or simply *prior*), and the term *p*(**d**∣**m**) is the likelihood function, which measures the accordance of the model to the new observations (technically *the conditional probability of the data given the model*). The term in the denominator on the right side of the equation, *p*(**d**), is called the *evidence* and represents the integral of the PPD over the prior probability density function:

(2) p(d)=∫p(m)p(d∣m)dm.

Basically, it represents a measure of the likelihood of the parameterization to the new data, and, thus, it can be used to compare different parameterizations of the physical model (Sambridge et al., 2006). The Bayesian approach can be really attractive in case of highly nonunique inverse problems, where deterministic solutions would be impossible or lead to unrealistic uncertainty estimates for the model parameters (Tarantola, 1987).

The PPD contains all the information we need about the investigated parameters, but its representation in a multidimensional space could be hardly displayed. Common estimators can be selected to make such information more readable and interpretable (Bodin, Sambridge, Rawlinson, et al., 2012). Those quantities follow the general form of a marginal probability of the PPD:

(3) Φ=∫f(m)p(m∣d)dm.

### 1.2 Parsimonious Parameterizations and Trans‐D Algorithms

Classical algorithms for performing Markov chain Monte Carlo (McMC) sampling do involve a fixed number of unknowns in each sampled model, that is, the model vector has fixed length while its components have variable values. In many cases, this means that the model has fixed resolution imposed by the user (e.g., fixed number of layers in 1‐D parameterization and of fixed number of degrees and orders in harmonic representations). Due to the fact that we are interested to define decoupled structures, but without introducing additional structures not supported by data, it is suitable to introduce trans‐D sampling, where the number of unknowns is an unknown itself (also known as reversible‐jump McMC algorithm [RjMcMC]; Green, 1995; Sambridge et al., 2006, 2013). Such algorithm can be used to sample models with a different number of parameters (i.e., the model vector has no more a fixed length), and it has been used to relax the constraint of an user‐defined resolution in single inversions, for 1‐D profiles (e.g., Dettmer & Dosso, 2012), 2‐D maps (e.g., Bodin, Sambridge, Rawlinson, et al., 2012), and 3‐D volumes (Piana Agostinetti et al., 2015). Trans‐D algorithms have been proved to perform a “parsimonious” sampling of the model space, that is, preferring a low‐dimensional model over high‐dimensional ones, if they can both explain the observed data at the same level (Malinverno, 2002). This behavior turns out to be the keystone for an algorithm, like ours, that needs to independently define, in a joint inversion, the coupled and decoupled portions of the structures. In fact, a model with fully coupled structures has lower model dimensions than a model with fully decoupled structures (by definition). At the same time, in the case where two properties are not resolved at the same scale, decoupling the structures may be needed to reduce the number of parameters for the poorly resolved property. In this way, decoupling can be used to get a simpler and more parsimonious model.

As we shall see with the synthetic examples presented below, adopting a parsimonious algorithm helps in discriminating which portions of the structures should be decoupled, based only on the amount of information contained in the two data sets.

## 2 An Algorithm for Defining Decoupled Structures

Defining decoupled structures can represent a significant advantage in the joint interpretation of two or more observables relative to the same investigated volume. On one side, when total spatial (or temporal) coupling between two model parameters is assumed in widely used joint inversion schemes (e.g., Gallardo & Meju, 2004; Heincke et al., 2017; Li & Qian, 2016), structures belonging to weakly resolved physical properties can be misinterpreted. On the other hand, when results from two independent inversions (each one focused on one physical property) are compared, local coupling of the structures is only suggested based on interpretation of the single images (e.g., Meju et al., 2003). Here we present a new algorithm that can be used to make statistical inferences about coupled or decoupled structures. The algorithm is developed in a Bayesian framework, and PPDs are numerically estimated using a McMC sampling of the model space (Mosegaard & Tarantola, 1995). A trans‐D algorithm sits in the core of the McMC sampling for proposing models with different degree of coupling, from fully coupled models to decoupled ones. Trans‐D algorithms allow to propose and compare models with a different number of dimensions (Malinverno, 2002) and, thus, they are suitable for comparing models with different parameterizations (Sambridge et al., 2006). A set of rules, so called *recipe*, is designed for an effective sampling of the model space. Those rules (also called *moves*) are randomly applied to the current model along the Markov chain for proposing a candidate. The acceptance probability of the candidate model is computed using a generalized Metropolis' rule (Gallagher et al., 2009). Thanks to the trans‐D behavior of the algorithm, our approach can move across completely different parameterizations and models, and it is able to reconstruct complex PPD using importance sampling. In the following, the algorithm is illustrated in details for a parameterization which encompasses two different physical properties, *N*_prop_=2, which is the minimum for a joint inversion of two different observables that put constraints on two physical properties. The recipe presented in this paper is straightforward to be adapted to two physical properties and an arbitrary number of observables. In Appendix A, we also depict how to extend the algorithm to investigate a model with *N*_prop_>2 physical properties.

### 2.1 Model Parameterization

In this study, a model is defined as an ensemble of a variable number of interfaces (Figure 1b). At each interface are assigned one or two physical properties considered to be related to the layer above the interface. In this way, we define three different classes (or *qualities*) of interfaces: interfaces with a change in both physical properties (quality 1 or *Q*_1_); interfaces with a change in the first physical property only (quality 2 or *Q*_2_); and interfaces with a change in the second physical property only (quality 3 or *Q*_3_). For each physical property, layering is defined by all the interfaces which own such physical property, that is, by all the *Q*_1_ interfaces and all *Q*_2_ interfaces for the first physical property and by all the *Q*_1_ interfaces and all *Q*_3_ interfaces for the second physical property (Figure 1b). Using this description, each model completely defines two 1‐D profiles for both the physical properties, where the layering in the two profiles can be partially different, that is, partial decoupling. Along these lines, a model can be formally written as

(4) $$m=(k_{1},k_{2},k_{3},z_{1},z_{2},z_{3},\alpha ,\beta ,\alpha_{H},\beta_{H},\pi_{\alpha },\pi_{\beta },),$$

where *k*_*i*_ is the number of interfaces of *i*th quality, *z*_*i*_ is the *k*_*i*_ vector of the depths of the interfaces of *i*th quality, **α** is the vector of length *k*_1_+*k*_2_ containing the values of the first property, α, and **β** is the vector of length *k*_1_+*k*_3_ containing the values of the second property, β. α_*H*_ and β_*H*_ represent the value of α and β for the half‐space.

In addition to the parameter related to physical properties, we also add the two hyper‐parameters *π*_*α*_ and *π*_*β*_ following the Hierarchical Bayes approach, for scaling the Covariance matrix of the error in the two data sets (Bodin, Sambridge, Tkalcic, et al., 2012).

### 2.2 Priors

Defining appropriate prior probability distributions, or simply *priors*, is a critical point in Bayesian algorithms (Backus, 1988; Efron, 2013). In this study, we use uniform priors for all the physical parameters and hyper‐parameters involved in the algorithm, that is, we set a minimum and a maximum allowed value for each parameter. Uniform priors can be easily handled and help to keep the algorithm simple, but more complex priors can be introduced if needed, without altering the theoretical framework of the structure decoupling (SD) algorithm. Given the approach adopted (see section 2.3), any kind of prior information can be introduced without the need of complex modification of the algorithm. Here the total number of interfaces is not explicitly parameterized. Instead, we separately parameterize the number of interface of each class (see section 2.1). Thus, for example, in our case the prior for total number of interfaces is not uniform, as it is given by the distribution of a sum of three uniformly distributed variables. Details on the prior distributions used in our tests can be found in sections 3.1.1.

### 2.3 Sampling Strategy, Likelihood Function, and Acceptance Probability

Different McMC implementations can be designed to efficiently sample a probability distribution. In this study, we adopt the approach of Mosegaard and Tarantola (1995) which is based on the Metropolis‐Hastings algorithm (Metropolis et al., 1953; Hastings, 1970), also called *extended Metropolis*. Mosegaard and Tarantola (1995) sample the PPD in two steps: first, a candidate model **m**_cand_ is proposed from the prior probability distribution, following a random walk especially designed for sampling the prior distributions. In the second step, the candidate model is accepted or rejected according to the ratio between the likelihood of the candidate model and the likelihood of the current model **m**_cur_. In our algorithm, the likelihood of a model **m** is computed as

(5) $$L(m)=p(d|m)=\overset{N_{\mathrm{prop}}}{\underset{i=1}{\prod }}\frac{1}{{\left[{\left(2\pi \right)}^{N_{i}}|C_{e,i}|\right]}^{1/2}}\exp\left(-\frac{1}{2}e_{i}^{T}C_{e,i}^{-1}e_{i}\right),$$

where *N*_*i*_ is the number of data points in the *i*th data set, and *C*_*e*,*i*_ is the Covariance matrix for the errors in the *i*th data set. *e*_*i*_ is the error vector defined as *e*_*i*_ = *d*_*i*_ − *g*_*i*_(**m**), where *d*_*i*_ is the measured *i*th data vector and *g*_*i*_(**m**) is the vector of the predicted *i*th data set by the model **m**. Following the approach in Mosegaard and Tarantola (1995), it turns out that the acceptance probability of each new candidate is

(6) $$\alpha =\min[1,L(m_{\mathrm{cand}})/L(m_{\mathrm{cur}})],$$

if the determinant |**J**| of the Jacobian matrix of the transformation from **m**_cur_ to **m**_cand_ is equal to 1. This Jacobian term is important when the dimensions of the vectors **m**_cur_ and **m**_cand_ are different. Thus, the construction of candidate models needs to be done with care to meet the condition on the determinant |**J**|. If such a condition is fulfilled, the candidate is always accepted if *L*(**m**_cand_)≥*L*(**m**_cur_). On the other hand, if *L*(**m**_cand_) < *L*(**m**_cur_), the random walk moves to the candidate model with probability equal to *L*(**m**_cand])/*L*(**m**_ _cur_).

The approach described above, for computing the acceptance probability *α* of a candidate model, differs from other implementation of RjMcMC algorithms, where such probability is computed from the general formulation (e.g., Gallagher et al., 2009, Equation 11):

(7) $$\alpha =\min\left[1,\frac{q(m_{\mathrm{cur}}|m_{\mathrm{cand}})}{q(m_{\mathrm{cand}}|m_{\mathrm{cur}})}\;\frac{p(m_{\mathrm{cand}})}{p(m_{\mathrm{cur}})}\;\frac{L(m_{\mathrm{cand}})}{L(m_{\mathrm{cur}})}\;|J|\right],$$

where *p*(**m**) is the prior distribution, *q*(**m**_cand_|**m**_cur_) is the proposal distribution of **m**_cand_ when the current model is **m**_cur_, and |**J**| is the determinant of the Jacobian matrix of the transformation from **m**_cur_ to **m**_cand_.

Here the acceptance probability *α* depends on a the proposal function *q*(**m**_cand_|**m**_cur_) defined by the user and its reverse *q*(**m**_cur_|**m**_cand_). In this case, the proposal distribution helps in tuning the acceptance ratio of each move to a good level (i.e., at about 0.234; see Rosenthal, 2009). On the other side, it can be difficult to explicitly calculate the proposal ratio *q*(**m**_cur_|**m**_cand_)/*q*(**m**_cand_|**m**_cur_) for complicated moves such as when a new model parameter is added or removed. The proposal function for complex prior distribution can be even worse to write. In our case, following the approach developed by Mosegaard and Tarantola (1995), any kind of prior information can be easily included and tested in the algorithm without having to entirely rewrite the algorithm. The main drawback is that the acceptance ratio can be very low in case of highly informative data, that is, when the PPD is significantly different from the prior probability distribution. In such cases, sampling according the priors could lead to overly slow convergence toward and to overly low acceptance rate.

### 2.4 Hierarchical Bayes

In classical joint inversion of many observables, the total likelihood of one model is composed of different terms, one for each observable, and subjective weights are introduced to control the influence of each single observable on the final solution. We significantly reduce subjectivity following a Hierarchical Bayes scheme, where the errors on the single data set are not considered as perfectly known. In our implementation, the Covariance matrix for the *i*th data set *C*_*e*,*i*_ is written as

(8) $$C_{\text{e,i}}=10^{\pi_{i}}\times C_{\text{e,i}}^{*},$$

where $C_{\text{e,i}}^{*}$ is the original Covariance matrix estimated from the data, and the term 10^*π*^_*i*_ is a factor that scales all the elements of $C_{\text{e,i}}^{*}$. The value *π*_*i*_ is commonly called *hyper‐parameter* and is treated as an unknown (Malinverno & Briggs, 2004). This vector of hyper‐parameters ***π***, which is integral part of each model, operates on the data space contrary to all the other parameters in **m** that operate on the model space. The hyper‐parameter *π*_*i*_ combines contributions from data uncertainties and approximations in the forward solvers *g*_*i*_, but it also include the relevant weight of the *i*th data set in the Likelihood function (Bodin, Salmon, et al., 2012).

### 2.5 Candidate Selection

Defining the recipe, the procedure that randomly perturbs the current model *m*_*cur*_ and propose a candidate model *m*_*cand*_, is a key issue in McMC sampling, as poorly designed recipes can lead to a less efficient sampling of the model space increasing the number of models needed to be sampled along the chain (Mosegaard & Tarantola, 1995). In particular, “weak” moves, that is, where the synthetics generated from the candidate model do not differ substantially from the synthetics generated from the current model, can lead to very slow sampling of the model space. On the opposite, “strong” moves, where perturbation in the synthetics are relevant, can risk to keep the chain stuck in a local minimum for a long time. Moreover, in our case, the recipe needs to be defined such that the sampling between different parameterizations for different physical properties would be consistent with trans‐D rules and allowing for both structural changes (spatial or temporal) and perturbation in the properties and number of properties belonging to each interface.

**Figure 2:**
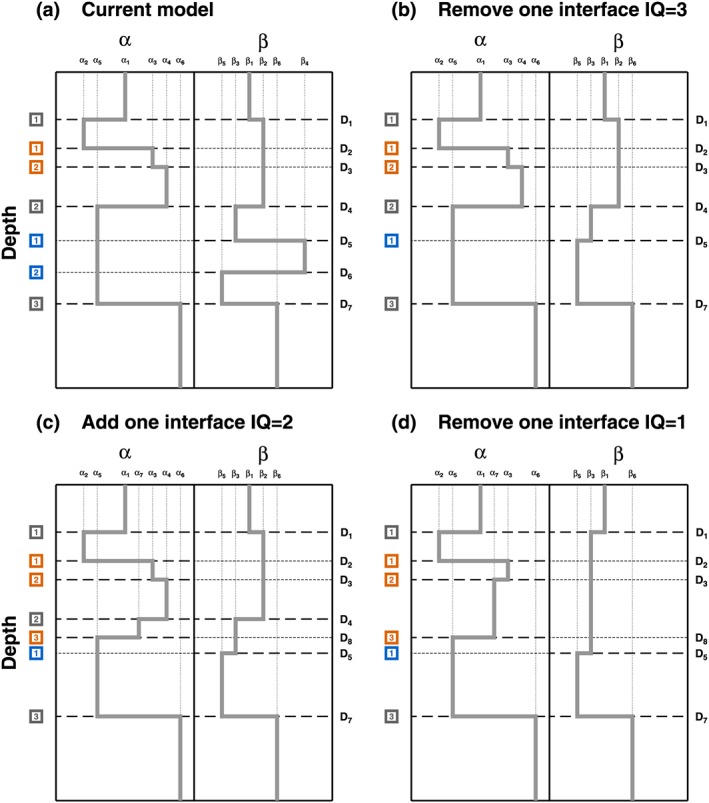
Example of models sampled along the Markov chain, following the recipe presented in this study. For sake of simplicity, model (b) is a perturbation of model (a), model (c) is a perturbation of model (b), and model (d) is a perturbation of model (c). This represents the situation where all candidate models are always accepted (i.e., prior sampling). (a) The “current” model (the same model presented in Figure 1b). Here the model is composed of seven interfaces. (b) First candidate model obtained removing one interface of “quality 3” (i.e., move 19 in our recipe: an interface with associated a value of β only). In this case, the β profile is updated, while α profile remains unchanged. (b) Next candidate obtained adding one interface of “quality 2” to the model presented in (b), move 16 in our recipe. Conversely to (b), here the α profile is updated, while β profile is left unmodified. (d) Finally, a candidate model obtained removing an interface of “quality 1”, move 15 in our recipe. Here both profiles are updated. Examples (b) and (c) illustrate some of the possible perturbations to a current model obtained using our recipe, where the two profiles are not updated simultaneously. More flexibility is given by moves 8–13 in our recipe, where profiles are perturbed with limited modification to the interface depths.

In this study, developed for *N*_prop_ = 2, our recipe is composed of 19 moves, where each move perturbs the model in some of its parameters. Details on each single move are given in Appendix A. The recipe is composed of three blocks of moves, grouped according to their potential perturbation of the current model structure, from low‐grade perturbation (first block), where only physical properties are perturbed, to high‐grade perturbation (third block), where both properties and structures are changed (Figure 2). The first block (moves 1–7) deals with the perturbation of the physical properties and hyper‐parameters, following a random walk that samples their prior probability distributions (see Appendix A in Piana Agostinetti & Malinverno, 2010, for details). The third block (moves 14–19) refers to the “classical” trans‐D 1‐D algorithms, where an interface is either added or removed from the model (e.g., Mandolesi et al., 2018). In this case, both the spatial (or temporal) structure *and* the physical properties are varied. Here three different classes of interfaces are present so six moves are needed. The second block of moves (moves 8–13) still groups trans‐D moves, in the sense that the number of dimensions varies between **m**_**cur**_ and **m**_**cand**_ (i.e., $\dim(m_{cur})\neq \dim(m_{cand})$), but here the number of interfaces *or* the number of physical properties are changed (while in the third block, both of them are perturbed). Moves belonging to the second block, not present in classical trans‐D algorithms, can be added to the recipe thanks to the potential existence of two different structures for the two different properties. These moves stay in the middle ground between the first and the third blocks, in terms of degree of model perturbation, and can facilitate the exploration of the model space, as they are trans‐D moves, but with lesser impact on the candidate with respect to the moves of the third block. In fact, we find that our recipe samples the model space proposing a full spectrum of moves, from strong to weak moves (i.e., moves have very different acceptance rate), enhancing the exploration of promising regions and allowing to move efficiently across the model space.

Our algorithm is applied to 1‐D cases, and our parameterization and recipe are described in terms of *interfaces*. For consistency with previous studies, interfaces are called *changepoints* when 1‐D temporal structures are investigated. In 1‐D problems, the structure can be either parameterized with interfaces (changepoints), or Voronoi cells. Using interfaces produces a more efficient sampling of the model space, in case where nontrivial 1‐D structures have to be reconstructed.

## 3 Geophysical Joint Inverse Problems: Test Cases for the SD Algorithm

We illustrate how our SD algorithm works using three different test inversions. The first two inversions make use of synthetic measurements: the joint inversion of two time‐varying shear‐wave splitting parameters and the joint inversion of magnetotelluric (MT) data and receiver function (RF). The third inversion exploits field measurements: the DTR and electromagnetic measurements from a borehole. We select such three inversions as they represent two different cases in terms of data structure. In the first and last inversions, the two observables share the same data structure along the *t* axis and the same statistics for the data uncertainties. Moreover, it is a simple example of *changepoint problems*, that is, where series of data are inverted to find the occurrence of abrupt variations of the data values according to the statistics of the data uncertainty. In changepoint problems, the forward solver **g** is reduced to a simple functions (here a step function). In the second inversion, the data structure is completely different for the two observables. In fact, one is a *derivative* observable, that is, it depends on variation of a property in the investigated volume, while the other is an *integral* observable, that is, the observation is given by the average value of the property within a defined volume (in our case, the dimension of the volume depends on the observe frequency). Complex forward solver is needed in this case, for simulating the observed data sets, and different degrees of correlation in the error statistics are assumed. We consider the first test inversion as a simple toy problem for illustrating the algorithm and the others as examples of more realistic geophysical inversions.

### 3.1 Shear‐Wave Splitting Parameters: θ and Δt

Shear‐wave splitting is a common observation in seismic field measurements, especially during the recording of local events in seismic sequences (Crampin, 1978). Shear‐wave splitting consists in the separation of the S wave, generated by an earthquake, in two polarized components, fast and slow, during the S wave propagation across an anisotropic rock volume at depth. The measurement of the time delay Δt between the two components can give an idea of the intensity of anisotropy in rocks. At the same time, thanks to the polarization of the two waves, we can compute a fast direction θ which indicate the direction of propagation of the fast S wave component, which is related to the orientation of, for example, microfractures in the anisotropic rock volume (Crampin, 1978). For a given single seismic station, two time series of θ and Δt can be computed from recording of local events over a time period of, for example, months. Such time series have been analyzed for monitoring time variations of the seismic properties of the rocks (Lucente et al., 2010; Piccinini et al., 2015). Time series are routinely analyzed using the classical changepoint inversion scheme, where values of the observed parameter are approximate using stepwise function and the algorithm searches for the most probable location of abrupt changes in the observed data (e.g., Sambridge et al., 2013). Trans‐D algorithms have been applied to changepoint problems (Hopcroft et al., 2007; Gallagher, 2012) obtaining relevant results in estimating both the existence and the number of changepoints in observed time series. It is worth noticing that a similar algorithm can be used for locating changes in borehole measurements, that is, not only for time series but also for 1‐D profiles (e.g., Reading & Gallagher, 2013). Here we jointly invert the two time series for retrieving the time period where changes in the observations are decoupled. In fact, changes in time delay Δt can indicate an increase in fractures density or fractures dimension in a given rock volume (Piccinini et al., 2015), but if changes in Δt are coupled to variations of fast direction θ, it is more likely that S waves are traversing different rock volumes, and the time variations are only apparent.

We jointly invert the two time series of Δ*t* and *θ* applying our SD algorithm. Errors on the measurements of the splitting parameters are considered uncorrelated; thus, a diagonal Covariance matrix is used. The values of the errors associated to the splitting parameters are set similar to those measured during field surveys (Piccinini et al., 2015): errors on Δ*t* vary between 0.003 and 0.017 s, with an average of 0.007 s, while errors on *θ* span between 6^∘^ and 54^∘^ with an average of 14^∘^.

**Figure 3:**
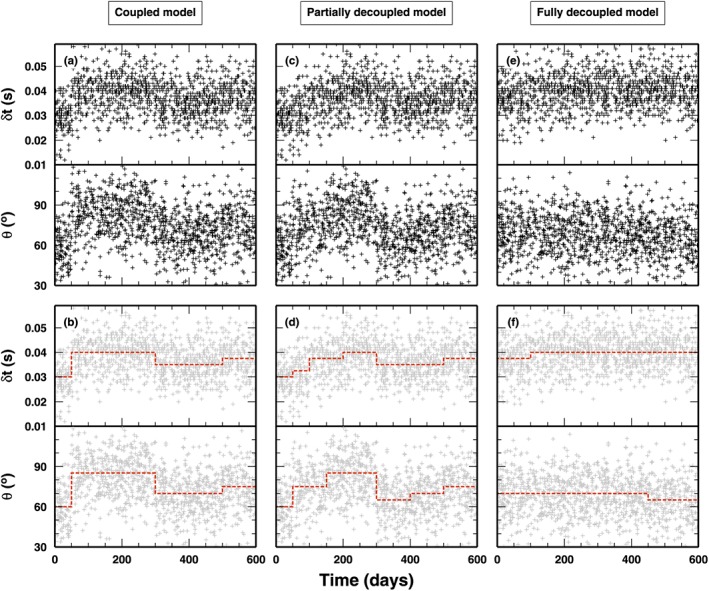
Synthetic data for the first test case: shear‐wave splitting parameters. To better illustrate how the algorithm works, we tested it using three different synthetic models. (a) Synthetic data computed using the coupled model shown in (b) as a red‐dashed line. (c) Synthetic data computed using the partially decoupled model shown in (d) as red‐dashed line. (e) Synthetic data computed using a fully decoupled model shown in (f) as a red‐dashed line.

Computing the forward solution for changepoint problems is trivial, as synthetic measurements are generally computed using a stepwise function of the observed parameter (Figure 3) or other simplified function (Gallagher, 2012). To remove a source of uncertainty from the inverse problem, we uniformly distributed the “observed” data in our synthetic test along the time axis. We acknowledge that such distribution does not mimic realistic data sets of splitting measurements, because natural seismicity tends to cluster in time. Instead, our choice reflects the need of a simple test case for our SD algorithm. More complex data distribution will be tested in the future.

#### 3.1.1 Prior Information

We select uniform prior probability distributions for our first inversion. Uniform distributions can be easily handled and strongly limit subjective selection of priors which can be viewed as a way to influence the solution of the inverse problem (Roy & Romanowicz, 2017). Here the number of *Q*_1_ changepoints is comprised between 1 and 30, while the number of changepoints in *Q*_1_ and *Q*_2_ classes are limited between 0 and 10 each. Δ*t* and *θ* can be varied between 0 and 0.1 s and 0^∘^ and 180^∘^, respectively. We acknowledge that *θ* is a circular variable, and our uniform prior distribution introduces a nonexistent boundary between 180^∘^ and 0^∘^ . A more versatile system of coordinates, like vector decomposition along two normal axes, should perform better. However, for sake of simplicity, we keep a standard uniform distribution. Our model is completely defined by two more hyper‐parameters *π*_*θ*_ and *π*_Δ*t*_ which are used to scale all the entries on the diagonal Covariance matrix of the errors, following the Hierarchical Bayes approach. The scale factors for the two data sets are computed as 10^*π*^_*θ*_ and 10^*π*^_Δ*t*_. In our case, priors on the two hyper‐parameters are uniform between −1 and 1, indicating that the errors can range between 1/10 and 10 times the original value.

#### 3.1.2 Results

We use the first groups of synthetic tests to illustrate the performance of the SD algorithm considering different degrees of coupling between the two physical properties. The algorithm is applied to synthetic data sets created using three different changepoint models: a coupled model, where all changepoints belong to class *Q*_1_; a partially decoupled model, where some changepoints belongs to class *Q*_1_, some to class *Q*_2_, and the remaining to class *Q*_3_; and a fully decoupled model, where changepoints belong to either class *Q*_2_ or class *Q*_3_. Moreover, for comparison, we also perform the same joint inversions using a classical RjMcMC algorithm (i.e., where only *Q*_1_ changepoints are considered). In Figure 3, we report the three models, and the three data sets generated from those models used as input to the SD and classical RjMcMC algorithms. In Figures 3a, 3c, and 3e, the three data sets are shown for both Δ*t* and *θ* observables. In Figures 3b, 3d, and 3f, the data are overplotted with the models used to generate them. For sake of comparison, the models are composed of both tiny and huge variations in the physical properties (relatively to their standard deviations), and the *partially coupled* model contains all the changepoints belonging to the other two models: coupled and “fully decoupled.” Clearly, the synthetic data sets are made of the synthetic predictions obtained from such models, to which random noise has been added following the noise statistics described in section 3.1.

**Figure 4:**
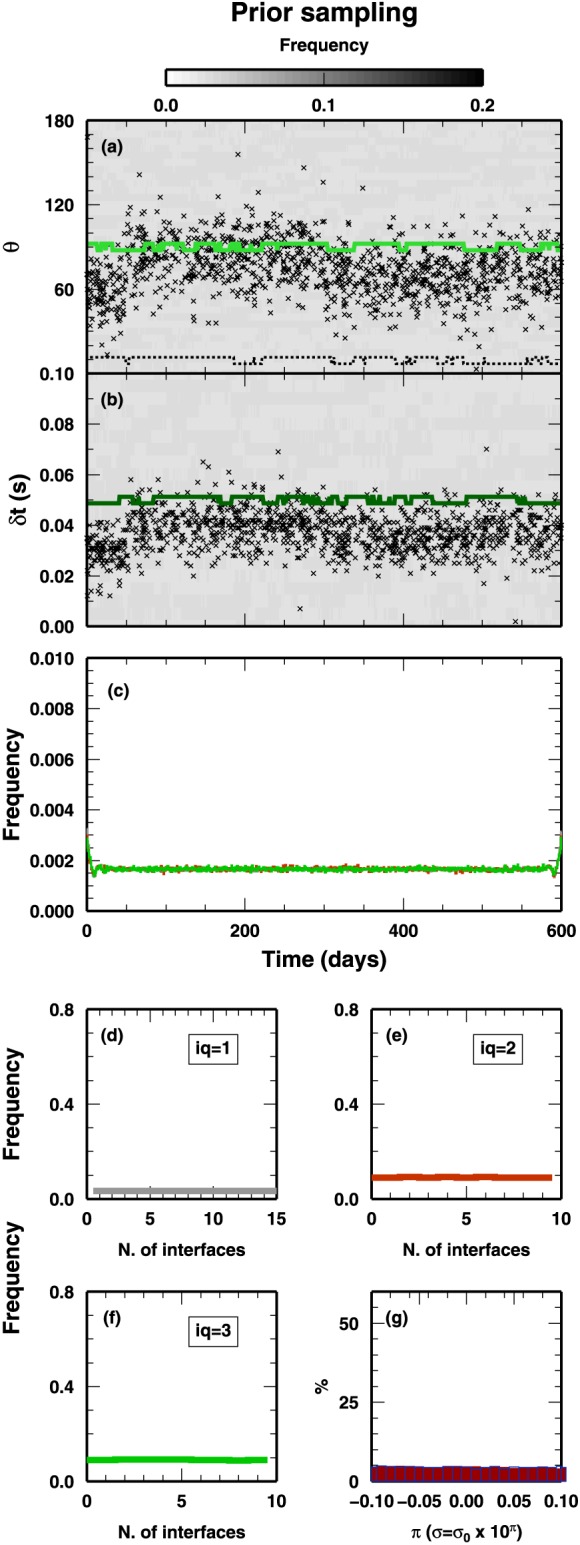
Prior sampling for the first test case. For all variables, uniform prior distributions are assumed and, thus, uniform posterior distributions are retrieved as expected. The θ (a) and δ t (b) properties. (c) The position of the interface along the x axis. Uniform priors for all classes of interfaces are retrieved. Red (class Q_2_) and gray (class Q_1_) lines are barely visible beneath the green line (class Q_3_). (d–f) The number of interfaces for each class. (g) The π parameters for the two data sets. The outlined histogram (error associated to δ t) is barely visible beneath the red histogram (error associated to θ).

For each test, we use 100 parallel chains, each chain sampling one million of models and discarding the models belonging to the first half of the sampling. Thus, we end up with 50 million models sampled from the PPD, and, for keeping the analysis more feasible and reduce models correlation, we select one every 100 of those models. Thus, our integrals are reconstructed using 5 × 10^5^ models. Given the trivial nature of the forward solver, in this test the SD algorithm takes less than 2 hr on a standard multicore laptop. In Figure 4, we show the results obtained when the algorithm is sampling models from the prior probability distributions (i.e., not considering the data and accepting all sampled models). This operation is useful to check if the algorithm is working properly and, more important, if implicit prior constraints have been introduced (e.g., Figure S6 in Chai et al., 2017). In our case, samples collected during this first test are distributed according to the previously described priors, certifying that our recipe is correctly implemented. In particular, prior uniform distribution for the number of changepoints in each class is retrieved, indicating that no preference is given to a predefined structure (coupled or decoupled).

##### 3.1.2.1 Coupled Model

**Figure 5:**
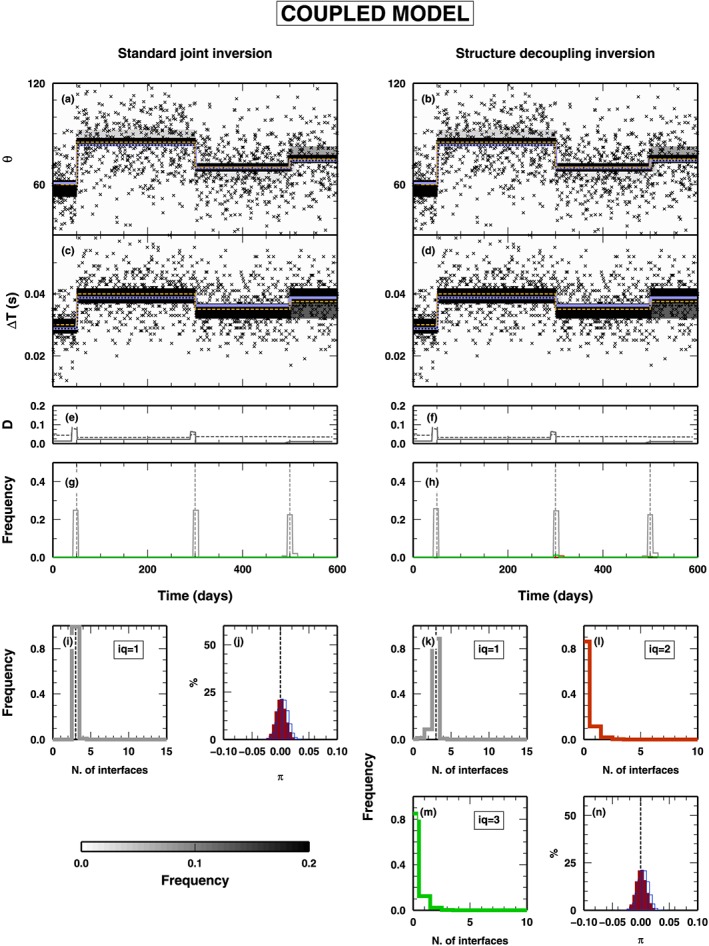
Results for the inversion of synthetic data presented in Figure 3a (fully coupled model). PPD for all the inverted parameters are shown. The panels on the left represent the results obtained using a standard joint inversion (i.e., using changepoints belonging to Q_1_ only). On the right, the panels show the results obtained with the structure decoupling algorithm. (a–d) PPD for the θ and δ t parameters. Gray‐scale colors display the PPD. Blue lines indicate mean posterior model; orange‐dashed lines indicate the true model. Black crosses show the inverted data. (e–f) Squared distance between true and mean posterior models, for θ (gray line) and δ t (gray‐dashed line). (g–h) PPD for the position of the changepoints in time. Histograms for changepoints of class Q_1_ (gray), Q_2_ (red), and Q_3_ (green). (i, k, l, and m) PPD for the number of changepoints. Histograms for changepoints of class Q_1_ (gray), Q_2_ (red), and Q_3_ (green). (j and n) PPD for the π parameters for the two data sets: δ t (outlined histograms) and θ (red histograms). PPD = posterior probability distribution.

In Figure 5, we illustrate the results of the synthetic test using an input data set obtained from a totally couple model, that is, where both *δ* *t* and *θ* vary at the same time. The panels on the left refer to the application of a classical RjMcMC algorithm, while the panels on the right report the application of the new SD algorithm. In this case, the results obtained with the two algorithms are extremely similar. The SD algorithm also makes use of classes *Q*_2_ and *Q*_3_ changepoints, but it is very limited, and the changepoints belonging to class *Q*_1_ are predominant in the final ensemble of model extracted from the PPD. In this case, a simple‐coupled model is enough to fit the data, and the SD algorithm does not need to decouple the structure by adding extra parameters.

##### 3.1.2.2 Mixed Model

**Figure 6:**
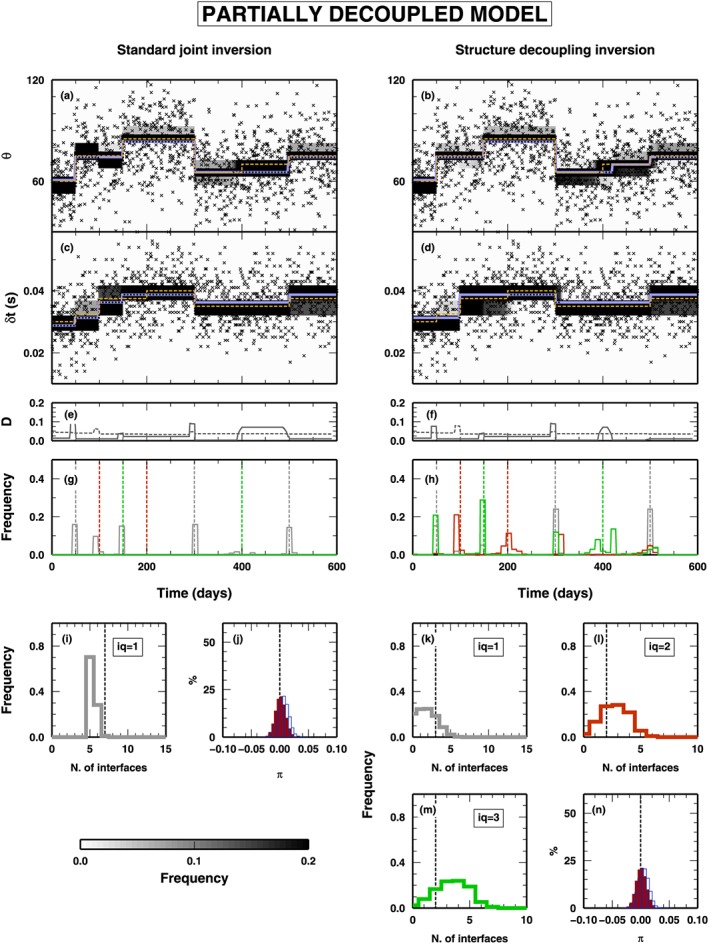
Results for the inversion of synthetic data presented in Figure 3b (partially decoupled model). See Figure 5 for symbol details.

In Figure 6, we show the results from the application of the two algorithms to a “mixed” data set, that is, computed from a model that contains all classes of changepoints. As in Figure 5, results from the application of the classical RjMcMC and SD algorithms are listed on the left and right columns, respectively. In both cases, the time evolution of the two properties are fairly well reproduced (Figures 6a–6d), even if, comparing the mean posterior and the true values of the two properties (blue‐ and orange‐dashed lines, respectively), the standard RjMcMC algorithm creates a fake discontinuity at *t* = 150 days in *δ* *t* and missed an changepoints at *t* = 400 days for *θ*. This is also evident, for *θ*, from the measure of the distance between the two models, mean posterior and true, reported in Figure 6e, where a relevant distance is found between *t* = 400 and 500 days.

Obviously, the two algorithms behave differently in sampling the time distribution of the changepoints (Figures 6g and 6h). The classical RjMcMC algorithm can use only class *Q*_1_ changepoints and substitutes the changepoints belonging to class *Q*_2_ and *Q*_3_, contained in the true model, when such changepoints are associated to strong variations in one of the two properties (changepoints at *t* = 100 and *t* = 150 days). Conversely, when such changepoints represent small variations of *δ* *t* or *θ*, the changepoints are not considered by the classical RjMcMC algorithm, due to its intrinsic parsimony (changepoints at *t* = 200 and *t* = 400 days). The SD algorithm samples a time distribution of the changepoints similar to the true one, in both changepoint classes and position. It is worth noticing that, at the locations of class *Q*_1_ changepoints in the true model (*t* = 50, *t* = 300, and *t* = 500 days), the SD algorithm inserts changepoints belonging to all classes, with a generally predominance of *Q*_1_ changepoints, not clearly indicating at which class belongs such changepoint. On the other hand, in presence of *Q*_2_ and *Q*_3_ changepoints in the true model, the algorithm definitely indicates which is the most probable “nature” of the changepoint found, inserting a clearly predominant class.

As expected from what observed above, the PPD of the number of changepoints, for the classical RjMcMC, has a modal value at 5, which is different from the sum of all changepoints in the true model (7), and few models were sampled with the correct number of changepoints (Figure 6i). The SD algorithm also preferably samples models with a smaller number of *Q*_1_ changepoints as expected, but it compensates that allowing for a larger number of changepoints in classes *Q*_2_ and *Q*_3_ (Figures 6k–6m). Finally, the PPDs for the hyper‐parameters related to the noise in the two data sets are similar between the two algorithms (Figures 6j and 6n), indicating that both data sets contribute correctly to the final solution.

##### 3.1.2.3 Decoupled Model

**Figure 7:**
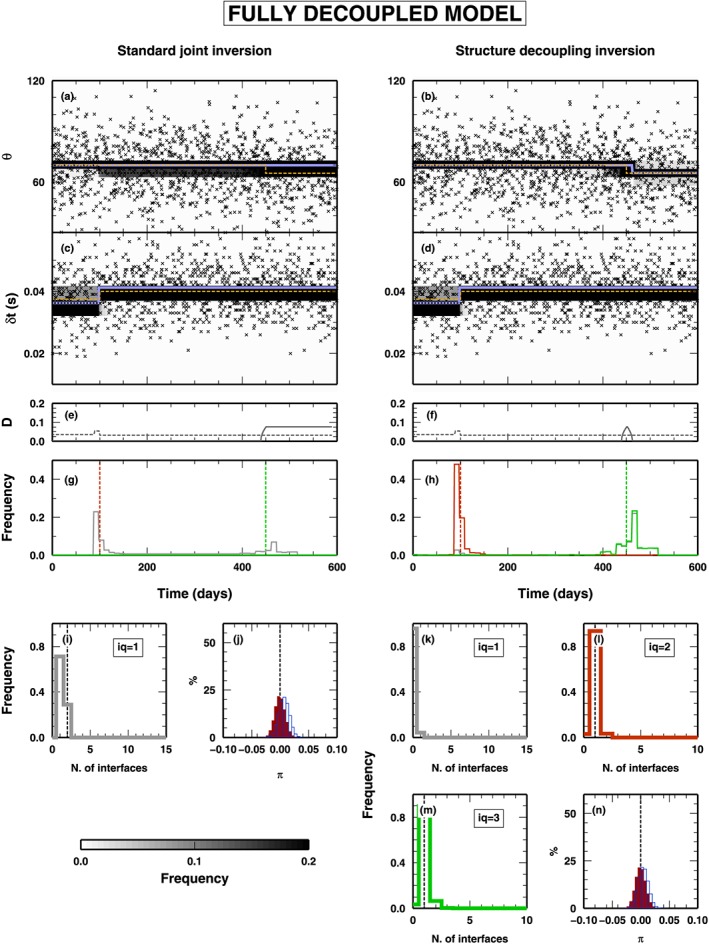
Results for the inversion of synthetic data presented in Figure 3c (fully decoupled model). See Figure 5 for symbol details.

The last test using the *δ* *t* and *θ* case study involves a true model composed on *Q*_2_ and *Q*_3_ changepoints only (Figure 7). As in Figure 5, results from the application of the classical RjMcMC and SD algorithms are listed on the left and right columns, respectively. In this case, the classical RjMcMC algorithm is not able to reproduce the variation in *θ* at *t* = 450 days, due to the limited variation of *θ* across such changepoint. Conversely, where the true model displays a relevant variation in *δ* *t* (at *t* = 100 days), the classical RjMcMC algorithm inserts a changepoint of *Q*_1_ class, without any appreciable variation in the PPD for *θ* other than an increase in the PPD dispersion around the mean value. The SD algorithm correctly reproduces both the time variations of both two properties. It is worth noticing that the SD algorithm makes use of *Q*_1_ changepoints as well but in a limited number (i.e., less than 10% of the models sampled from the PPD contain an changepoint in *Q*_1_ class; see Figure 7k).

For the classical RjMcMC algorithm, given the mismatch between true and mean posterior model, the distance between the two models is very large (between *t* = 450 and 600 days), while it is limited in the case of the SD algorithm (Figures 7g and 7h). The discrepancy observed between the true and the mean posterior model sampled by the classical RjMcMC algorithm is not reflected in the PPD of the hyper‐parameters, where both algorithms seem to give similar results, for both observables (Figures 7j and 7n).

### 3.2 MT and RF Data: ρ and V_S_

MT and RF analyses are two widely applied geophysical techniques aimed to reconstruct subsurface structures beneath an isolated MT and seismic stations, respectively. MT data are often used to image 2‐D and 3‐D structures, but they can be used for 1‐D modeling under precise conditions (i.e., symmetry of the Impedance tensor). In general, both MT and RF data are inverted for retrieving a 1‐D model beneath the station, in term of resistivity and *S* wave velocity, respectively. Both observables have been inverted independently using a RjMcMC algorithms (Mandolesi et al., 2018; Piana Agostinetti & Malinverno, 2010). Given the different “structure” of the MT data set with respect to the RF data set, they have been jointly inverted to better exploit the potential of seismic data in constraining seismic interfaces at depth (Le et al., 2016), with the implicit assumption that the two models would share the same interfaces (Moorkamp et al., 2007). In fact, RF data set consist in a band‐limited time series, which is a function of the gradient of the *S* velocity at depth (i.e., it is a “derivative” quantity, meaning that it is sensitive to the changes of the quantity at depth). On the contrary, MT data can be expressed in values of the Impedance tensor, which are “integral” quantities because they depend on the average resistivity value of a rock volume (where the dimension of the volume depends on the frequency observed). The complementary nature of the two data sets make them unbalanced candidates in a joint inversion, if we impose a fully coupled structure to the two physical properties. In fact, due to the limited sensitivity to interface locations in MT data (Mandolesi et al., 2018), in a joint inversion, the layering would be surely dictated by the RF data set.

Here we apply our algorithm for finding the portions of the two 1‐D profiles (in resistivity and *S* wave velocity) which display coupled (or decoupled) structures. As a consequence of the different data structure for the two data sets, in this test case, we consider two different uncertainty models for MT and RF data. As generally assumed in literature, errors associated to MT measurements are considered uncorrelated and estimated equal to a fixed fraction of the measurement. Thus, they can be represented using a diagonal matrix where, for the *i*th datum $d_{\mathrm{MT}}^{i}$, we have $\sigma_{\mathrm{MT}}^{i}=0.05\times d_{\mathrm{MT}}^{i}$. On the other hand, RF is a band‐limited time series, and a full correlation matrix must be used to model the associated uncertainties. Here we follow the approach described in Bodin, Sambridge, Tkalcic, et al. (2012), and we assume an exponential Covariance matrix with a fixed correlation length (*r* = 0.85) and variance of the data noise ( $\sigma_{\mathrm{RF}}^{i}=0.025$). Forward solutions are computed following the propagation matrix approach described in Mandolesi et al. (2018) for MT data and Shibutani et al. (1996) for the RF data. Such approaches allow to compute a forward solution in a fractions of second, which are necessary to obtain in the order of millions of models in reasonable amount of time.

#### 3.2.1 Prior Information

Uniform prior probability distributions are selected also for our second inverse problem. Here the number of *Q*_1_ interfaces is comprised between 1 and 40, while the number of interfaces in *Q*_2_ and *Q*_3_ classes are limited between 0 and 40 and 0 and 10, respectively. Interfaces depth follows a uniform prior between 0‐ and 60‐km depth. For the physical properties, we adopted a uniform distribution between 1.5 and 4.0 Ω*m* for the logarithmic value of the resistivity *ρ*, while the uniform prior is bounded between 2.5 and 5.0 km/s for the *S* wave velocity *V*_*S*_. Even in this case study, the two hyper‐parameters, *π*_MT_ and *π*_RF_, that are used to scale the two Covariance matrices, can vary between −1 and 1. The Covariance matrix for the MT data set is, thus, a diagonal matrix multiplied by 10^*π*^_MT_. For the RF data set, the Covariance matrix can be expressed as $C_{E}^{\mathrm{RF}}=10^{\pi_{\mathrm{RF}}}\times C_{\mathrm{RF}}^{*}$, where $C_{\mathrm{RF}}^{*}$ is the exponential correlation matrix computed using correlation length *r* = 0.85 and variance of the data noise $\sigma_{\mathrm{RF}}^{i}=0.025$ (Bodin, Sambridge, Tkalcic, et al., 2012).

#### 3.2.2 Results

**Figure 8:**
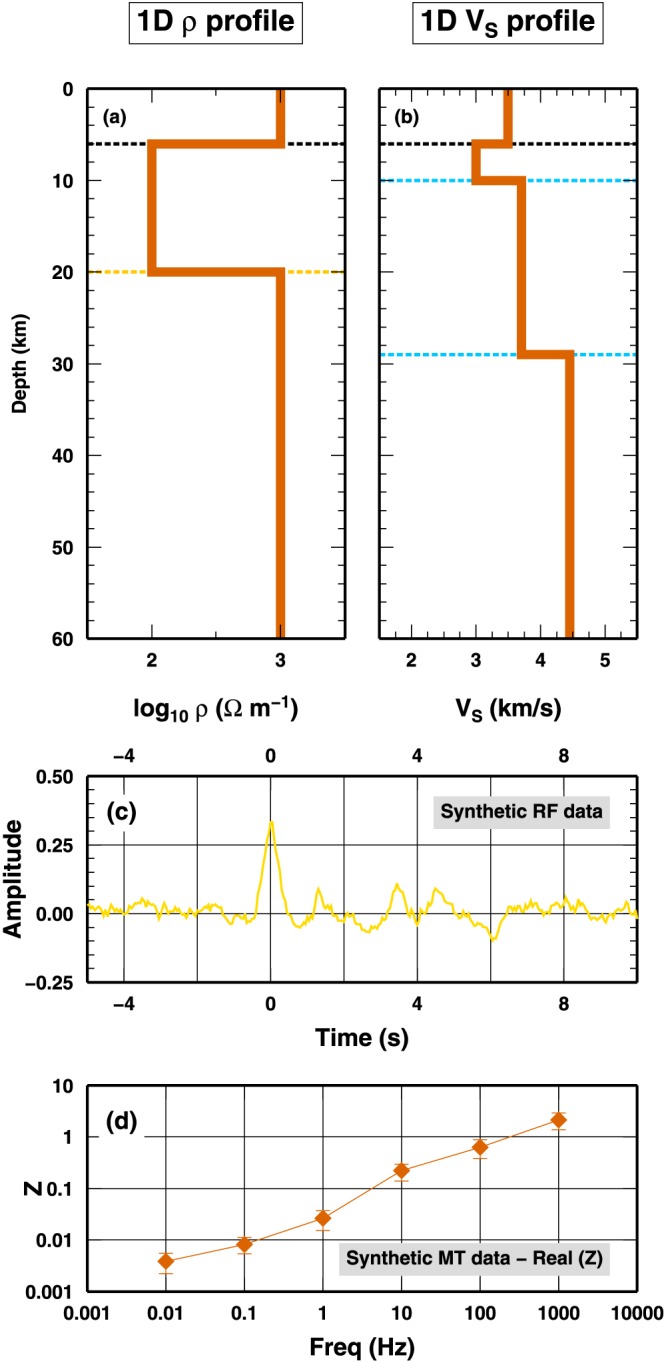
Synthetic models and data for the second test case: MT and RF data. (a and b) The ρ and V_S_ profiles. ρ profiles is composed of two interfaces: one interface in class Q_1_ and one interface in class Q_2_. The V_S_ profile is composed of three interfaces. One interface in class Q_1_ (the interface shared with the ρ profile) and two interfaces in class Q_3_. (c) Synthetic RF data generated using the V_S_ model in (b). (d) Synthetic MT data generated using the ρ model in (a). In both cases, we added noise to the synthetics as described in the text. MT = magnetotelluric; RF = receiver function.

We use the second groups of synthetic tests to illustrate the performance of three different algorithms on the joint inversion of MT and RF data: a fixed dimension McMC code, the classical RjMcMC approach, and the SD algorithm. The true model presents a combination of one *Q*_1_, one *Q*_2_, and two *Q*_3_ interfaces, and it mimics realistic Earth's crust properties as found along the Apulia continental margin (Amato et al., 2014; Patella et al., 2005; Piana Agostinetti & Malinverno, 2018). In Figure 8, we report the 1‐D resistivity and *S* wave velocity models and the data sets generated from those models, to which random noise has been added following the noise statistics described in section 3.2. In this case, the computation has been quite expensive in terms of computation resources, and the tests were executed on a Linux cluster. For each test, we ran 250 independent parallel chains, each sampling 4 millions of models, for a total number of models equal to 1 billion. Half of the models were discarded as part of the burn‐in phase. The PPD has been reconstructed collecting one every 1,000 models in the post burn‐in phase. The total computation time was around 12 hr, using 250 CPUs (one CPU per chain). The long computation time, as discussed below, would represent the main shortcoming of the proposed new approach.

##### 3.2.2.1 Fixed Dimension Algorithm

**Figure 9:**
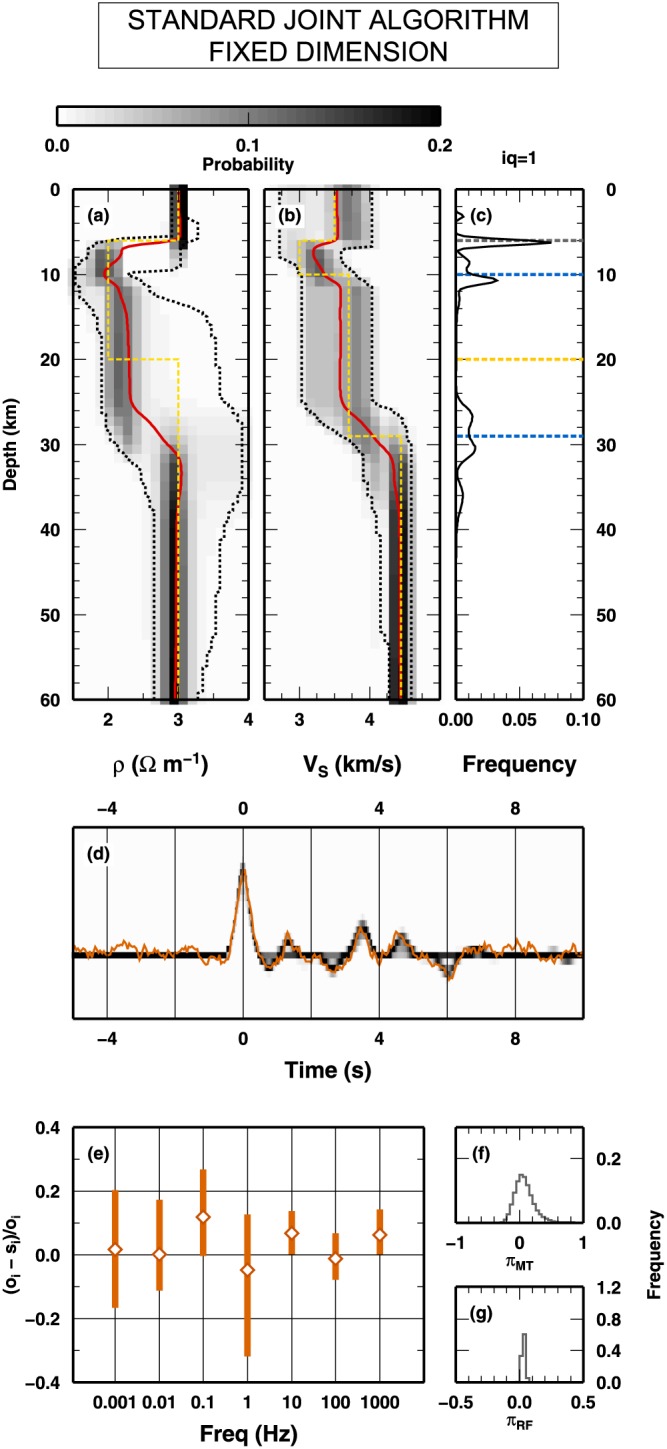
Results for the inversion of synthetic data presented in Figure 8 using a fixed‐dimensional algorithm. Here the number of interfaces is fixed to 3. Results are presented in term of PPDs. (a and b) PPD for the ρ (a) and V_S_ (b) properties. Gray colors represent probabilities. Yellow‐dashed lines indicate the “true” models. Red lines show the posterior mean models. Black‐dotted lines display 90% confidence level. (c) PPD for the depth of the four interfaces. Horizontal‐dashed lines indicate the position of the interfaces in the true model: gray = interfaces in class Q_1_, yellow = interfaces in class Q_2_, and blue = interfaces in class Q_3_. (d) Fit between RF data and prediction computed along the Monte Carlo sampling. A red line indicates the “observed” RF from Figure 8c. Gray colors indicate PPD if the synthetics. (e) Fit between observed and synthetic magnetotelluric data, expressed in terms of relative deviations form the observed value. The open diamonds represent the mean posterior relative deviation for each frequency; the red vertical bars indicate the 2σ interval for the posterior relative deviation. (f and g) PPD for the scale value of the errors for magnetotelluric data (f) and RF data (g). RF = receiver function; PPD = posterior probability distribution.

In the first case, a fixed dimension McMC algorithm is applied to the joint inversion of the MT and RF data presented in Figure 8. The fixed dimension McMC is developed from the SD algorithm, allowing the SD algorithm to use *Q*_1_interfaces only, and extracts samples from the PPD of the two properties at depth, *ρ* and *V*_*S*_. Here the number of interfaces is fixed to 3, which represent the number of interfaces in the *V*_*S*_model used to compute the synthetic observations. In Figure 9, we report the results of the inversion. In Figures 9a and 9b, the 1‐D marginal PPDs of the two properties are shown. While the distribution for *V*_*s*_ closely mimics the “true” model used to produce synthetic observations, the posterior *ρ* profile does not resemble the profile for *ρ* in Figure 8. Here, since the spatial variations in parameter *ρ* are less well resolved than *V*_*S*_, the coupled geometry is dictated by the geometry of *V*_*S*_, resulting in an overparameterization for *ρ*. In particular, it seems to include a spurious low‐resistivity layer between 10‐ and 30‐km depth. At such depth level, we observe that the posterior confidence intervals for *ρ* are larger than the intervals at shallow depth. The distribution of interfaces at depth, Figure 9c, shows that the depths of the main interfaces are all fairly reproduced, and additional interfaces are suggested, even if, looking to their associated frequency values, they seems to have low relevance. At the same time, both posterior synthetics and PPD of the scale parameter of the errors indicate that the sampled models coherently fit synthetics (differently, the scale factor would be significantly larger than 0.0).

##### 3.2.2.2 Trans‐D Algorithm

**Figure 10:**
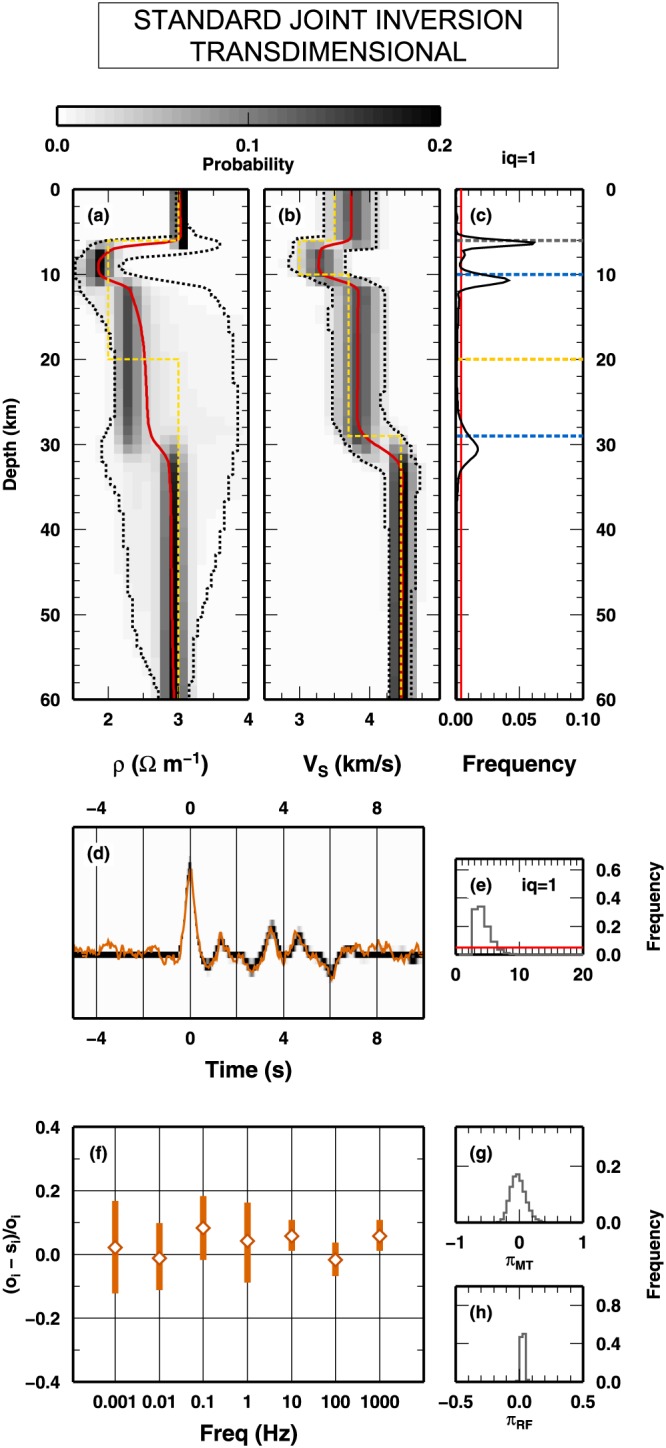
Results for the inversion of synthetic data presented in Figure 8 using trans‐dimensional algorithm. Results are presented in term of posterior probability distributions. Details for (a) to (d) as in Figure 9. (e) Posterior probability distribution of the number of interfaces. The red line indicates the uniform prior distribution. (f) as in (e) in Figure 9. (g) and (h) as in (f) and (g) in Figure 9, respectively.

In the second computation, the SD algorithm is reworked as a classical RjMcMC algorithm, where the number of interfaces can be changed along the Markov chain. As for the previous test, we here use *Q*_1_ interface only. The number of *Q*_1_ interfaces can vary from 1 to 20. The results obtained with the classical RjMcMC algorithm are presented in Figure 10. Similar to the previous test, the 1‐D marginal PPD for the *V*_*s*_ closely matches the true model, while, again, the 1‐D marginal PPD for *ρ* does not resemble that model. Here, again, the fully coupled geometry imposes an overparameterization to the less resolved parameter *ρ*, which results in an artificial low‐resistivity layer between 10‐ and 30‐km depth. The main difference from Figure 9 can be seen in panel c where we observe that the posterior distribution for the depths of the interfaces strongly support the presence of three interfaces at the depth levels of the *Q*_1_ and *Q*_3_ interfaces present in the true model. The fit between observed and synthetics is high, and the scale parameters for the uncertainty are close to the expected value (0.0).

##### 3.2.2.3 SD Algorithm

**Figure 11:**
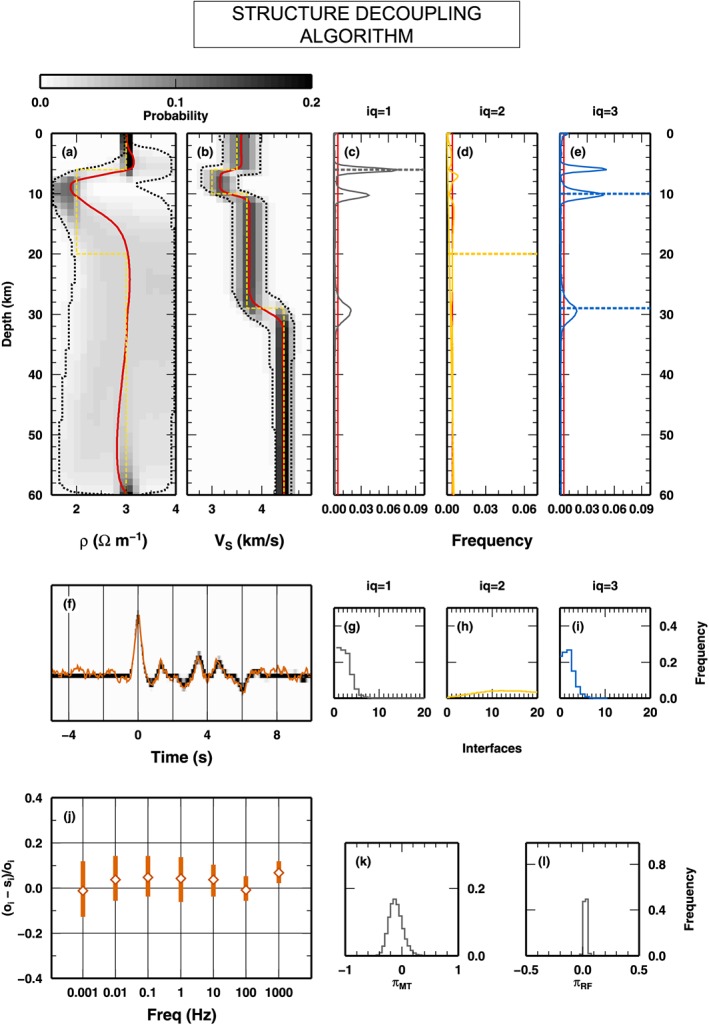
Results for the inversion of synthetic data presented in Figure 8 using the structure decoupling algorithm. Results are presented in term of PPDs. Details for (a) and (b) as in Figure 9. (d and e) PPD for the number of interfaces in class Q_1_ (c), class Q_2_ (d), and class Q_3_ (e). Red lines indicate the prior uniform distribution. Dashed lines represent the position of the interfaces in the “true” model. (f) as in (d) in Figure 9. (g–i) PPD of the number of interfaces in class Q_1_ (g), class Q_2_ (h), and class Q_3_ (i). (j) as in (e) in Figure 9. (k) and (l) as in (f) and (g) in Figure 9, respectively. PPD = posterior probability distribution.

We now test the SD algorithm on the data sets presented in Figure 8. Here the SD algorithm samples interfaces belonging to all classes, *Q*_1_, *Q*_2_, and *Q*_3_. The minimum number of interfaces in the three classes can reach 0 (for *Q*_2_ and *Q*_3_) and 1 (for *Q*_1_), that is, both fully coupled model and almost fully decoupled models are considered. The 1‐D marginal PPDs for the *V*_*S*_ and *ρ* are shown in Figures 11a and 11b. Again, the 1‐D marginal PPD for *V*_*S*_ closely follows the true model used to produce the synthetic observations. Differently from the previous tests, the 1‐D marginal PPD for *ρ* becomes almost undefined below about 15‐km depth, and it does not display the low‐resistivity layer at 10–30‐km depth. Here the parsimonious nature of the SD algorithm allows to decouple the structure at 10‐km depth, in order to remove a discontinuity in *ρ* that is not required by the data. This is an example where decoupling the structures enables to produce simpler models. The distributions of interfaces at depth within the three classes (Figures 11c–11e) indicate that the correct class of interfaces is used at the correct depth location. There is no relevant interfaces for *Q*_2_ interfaces (Figure 11d), as expected from the low resolution at depth of the MT data. It is worth noticing that at the depth of the shallowest interface, a *Q*_1_ interface in the true model, interfaces belonging to the other two classes, *Q*_2_ and *Q*_3_ interfaces, are also sampled, indicating that the algorithm can put there one *Q*_1_ interface or a combination of the other two classes. We observe, from the frequency values, that the most probable interface falls in the *Q*_1_ class. The same observation applies to the interface at 30‐km depth. Here the original *Q*_3_ interface, found in the true model, can be replaced by the algorithm with a *Q*_1_ interface. However, that *Q*_1_ interface, as seen from Figure 11a, should not place any constraint in the *ρ* parameter (i.e., its mean, and confidence levels, posterior value does not change from above to below 30‐km depth).

In Figures 11f–11j, we plot the PPD for the synthetic measurements (RF), relative error (MT), and number of interfaces in the three classes. The fit between observed and synthetic RF is high as in the previous test, and the relative errors on MT are smaller. The PPD for the number of interfaces for *Q*_1_ and *Q*_3_ classes, where strong constraints are present in the observations, are close to the true value, $n_{Q_{1}}=1$ and $n_{Q_{3}}=2$, respectively. For interfaces in class *Q*_2_, the PPD of the number of interfaces is close to the prior values, indicating the data do not contain information on an additional layering for *ρ*.

### 3.3 Borehole Logs: DTR and EAL

**Figure 12:**
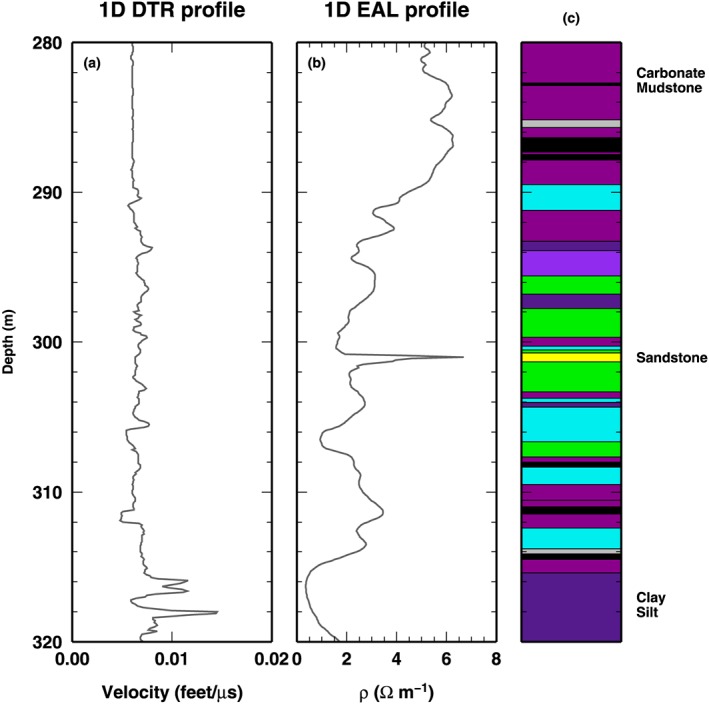
Borehole data used in the real‐world test case: reversed delay time (DTR) and electromagnetic array log (EAL). (a and b) The DTR and EAL data in the observed depth range (280–320 m). (c) Lithostratigraphy from the same borehole and depth range. Colors denote formations: coal = black, coaly shale = gray, carbonate mudstone = dark magenta; claystone = cyan, sandstone = yellow; silt = purple; dolerite = dark orange, and tuff = red. Names indicate the position of the formations cited in the main text.

We select two borehole logs to test our algorithm on field geophysical measurements. Such data sets have been used in Reading and Gallagher (2013) to investigate different components of uncertainties associated to borehole modeling. In Reading and Gallagher (2013), DTR and electromagnetic array log (EAL) in the depth section between 280 and 320 m are investigated, together with density and neutron logs, to define the characteristics of lithology interfaces and to measure the internal variability of the main formations. Here we limit our investigation to the first two observables, DTR and EAL. Such observables display well‐defined portions of the 280–320 m depth range where the profiles seems to be decoupled (Figure 12). Comparison with the borehole lithostratigraphy gives us the opportunity of discussing the retrieved coupled/decoupled sections of the profiles in term of boundaries between formations. In particular, a sharp increase of the value of EAL in the sandstone formation (about 300–302 m) is not reflected in any kind of variation in the DTR value in the same depth range. Conversely, at the end of our depth range, within the coal formation (about 310–316 m), the DTR value displays large variability, while EAL value shows a smooth increasing trend.

In this case study, the algorithm works as a simple “changepoint” regression (as in the first synthetic test), where we search for the family of most probable layered models that predict DTR and EAL data, considering constant DTR and EAL within each layer. This assumption, and the use of a RjMcMC algorithm, recalls the methodology used in Reading and Gallagher (2013), with the most interesting difference being, in our implementation, the potential for exploiting decoupled structures. The model is composed of a variable number of interfaces, belonging to the three different qualities, Q_i_,i = 1,2,3, where Q_1_ interfaces contain information about both DTR and EAL, while Q_2_ and Q_3_ interfaces represent DTR and EAL only, respectively.

Given the nature of the borehole measurements, we assume the data points of the two logs to have uncorrelated uncertainties and, thus, a diagonal Covariance matrix is used. Errors on the DTR and EAL data are arbitrarily set to 0.01 feet/μs and 10.0 Ω/m (about 3000 m/s and 10.0 Ohm/m), respectively. The Hierarchical Bayes approach is adopted to retrieve more realistic variance estimates.

#### 3.3.1 Priors

Priors on the investigated parameters are set uniform. The maximum number of changepoints, for each quality, is set to 100. A minimum distance of 0.1 m is considered between interfaces, as defined in Reading and Gallagher (2013). This choice excludes models where two interfaces fall in‐between two data points, because such layer would be invisible to the data. While this choice speed‐ups the algorithm, our tests indicate that it does not prevent the correct reconstruction of the PPD, as expected. Min/max intervals for DTR and EAL are set to [0;0.02] feet/μs and [0;8.0] Ω/m, respectively. A uniform prior is also defined for the two hyper‐parameters π_DTR_ and π_EAL_ which are used to scale the diagonal Covariance matrix of data errors, following the Hierarchical Bayes approach. The scale factors for the two data sets are computed as 10^π^_DTR_ and 10^π^_EAL_. In our case, priors on the two hyper‐parameters π_DTR_ and π_EAL_ are uniform between [−2.5;1], indicating that the errors can range between 3/1,000 and 10 times the original value.

#### 3.3.2 Results

The last test inversion illustrates how the SD algorithm performs on field measurements. The borehole logs exploited as observed data present different patterns of variability, at different depth ranges, that make such data valuable to highlight the performance of the algorithm in spotting potentially decoupled structures. The geophysical inverse problem is cast as a changepoint inversion. In this case, the computation is not CPU‐time intensive, and the tests were executed on a commercial laptop. We ran 100 independent parallel chains, each sampling 1 millions of models, for a total number of models equal to 100 millions. Half of the models were discarded as part of the burn‐in phase. The PPD has been reconstructed collecting one every 1,000 models in the post burn‐in phase. The full computation takes about 1 hr of CPU‐time.

**Figure 13:**
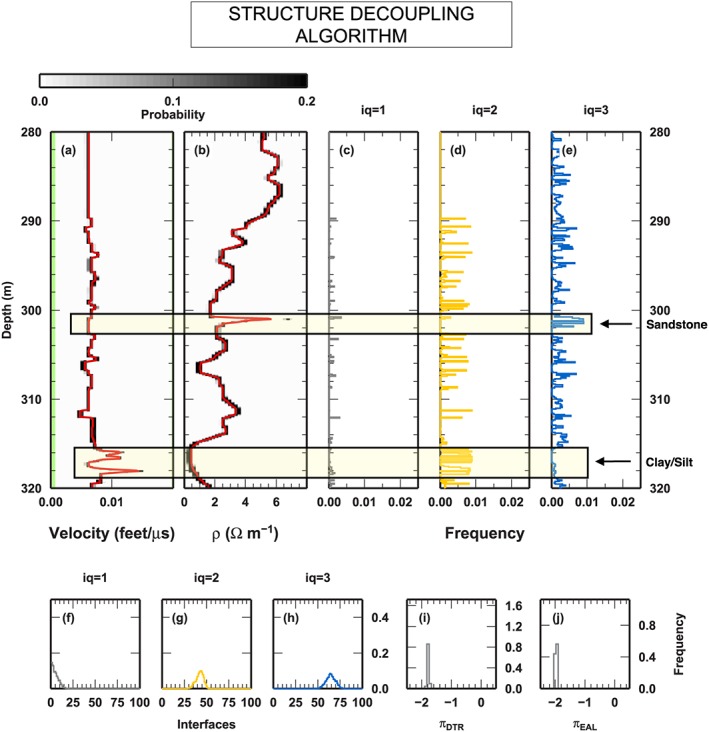
Results for the inversion of borehole data. In (a–e), the two red boxes indicated the depth range for the formations examined in the main text. (a and b) Posterior realization of the reversed delay time (a) and electromagnetic array log (b). Gray colors indicate probability. Red lines are mean posterior models. (c–e) Posterior distribution of the interfaces at depth for Q_1_ (c), Q_2_ (d), and Q_3_ (e). Posterior distribution for the number of interfaces for Q_1_ (f), Q_2_ (g), and Q_h_ (h). Posterior distribution for the scaling factor π_X_ of the uncertainties, where X is reversed delay time (i) or electromagnetic array log (j). Scaling factor is computed as 10^π^_X_.

The PPD of the investigated parameters, computed from the 50 millions models retained after the burn‐in phase, is shown in Figure 13. Posterior values for DTR and EAL closely resemble the values of the borehole logs (Figures 13a and 13b). The depth of the interfaces, for the three qualities, clearly indicates the predominance of one kind of interfaces with respect to the others at some depth levels (Figures 13c–13e). In particular, at the depth of the sandstone formation, the distribution at depth of *Q*_3_ interfaces show a maximum, where no interfaces are present for *Q*_1_ and *Q*_2_ qualities. At the depth range of the coal formation, at about 310–316 m, the smooth trend in the EAL measurements is reproduced with a limited number of *Q*_3_ interfaces, while reproducing the more complex, highly variable pattern in DRT value requires a consistent number of interface of *Q*_2_ quality. The larger low‐frequency variability in the EAL profile with respect to DTR profile is likely responsible of the large number of interfaces used for modeling such observation (Figures 13f–13h). In fact, the SD algorithm approximately uses between 50 and 75 *Q*_3_ interfaces, where it only puts between 30 and 50 *Q*_2_ interfaces. Remarkably, the number of *Q*_1_ interfaces is limited. Position of such *Q*_1_ interfaces should be investigated and compared to boundaries between formations, to highlight potential correlation between coupled structures and well‐defined lithological contact. For example, the Carbonate Mudstone (magenta) formation at about 312 m, sandwiched between Coal (black) and Claystone (cyan) formations, is defined by two sharp *Q*_2_ interfaces, but only the lower one has also a relevant *Q*_1_ interface, pointing out that Mudstone‐Claystone contact could be a relevant boundary for seismic velocity only (Figures 13a–13c). However, this kind of investigation is beyond the scope of the present study. Finally, we observe that the scaling parameters for the data uncertainties are both sampled around 10^−2^. This fact indicates that our assumptions on the data errors overestimate such values, as expected.

## 4 Discussion

We tested our algorithm on two different geophysical inverse problems using synthetic data: a changepoints problem aimed to locate variations in time of monitored parameters and a more complex geophysical inversion for retrieving the subsurface properties in terms of resistivity and seismic velocity. We applied to both problems a standard algorithm and the new SD algorithm, to highlight the advantages and shortcomings of our new approach with respect to more widely used RjMcMC methodologies. Overall, our new algorithm outperforms the classical‐fixed dimension and trans‐D McMC approaches, in case of decoupled structures. It better resolves structures when required by the data, without adding spurious artifacts due to overparameterization. That is, the SD algorithm correctly limits the information on the structure where the observables have limited resolution (e.g., Chave & Jones, 2012), and we demonstrate that the SD algorithm can point out tiny decoupled changes in one of the properties as seen in Figure 7b for the *θ* parameter.

The application of the SD algorithm to field measurements confirms the performance of the algorithm in distinguishing structures between two parameters, here DTR and EAL, at the depth levels of the 1‐D profile where the two observables show clearly different oscillations. Our application of the SD algorithm to the reconstruction of borehole measurements confirms the feasibility of the RjMcMC approach for characterizing the lithological interfaces in terms of changepoint locations, as done in Reading and Gallagher (2013) using a standard RjMcMC algorithm

As for many Monte Carlo algorithms, the main shortcoming of our approach is the computational time. Our model parameterization, which comprises three different set of interfaces, and our recipe, which encompasses three different blocks of moves for perturbing the current model, increase the computation time due to the enlarged model space and the need of testing all the different moves. The latter point is extremely relevant here, where we cannot a priori define which block of moves is the most efficient for ensuring both fast convergence to the global maximum of the likelihood and model space exploration. Different modifications to standard McMC sampling have been designed for increasing the exploration of the model space. In particular, our algorithm could be easily included into a more general Parallel Tempering (PT) scheme (see Appendix A in Sambridge, 2014, for a general PT work‐flow). Briefly, a PT scheme allows to keep a number of RjMcMC chains sampling a smoothed (i.e., tempered) version of the PPD, which guarantees such chains to sweep more broadly across the model space. This scheme has been proved to improve the sampling for complex PPD (Dettmer & Dosso, 2012; Dosso et al., 2012) in case on high nonlinearity and nonuniqueness, which is the case here, where three different sets of interfaces interact.

In this study, we adopt the approach designed by Mosegaard and Tarantola (1995), where the McMC algorithm is composed of two consecutive steps. First, a candidate model is proposed using a sampler that asymptotically samples the prior probability distribution. Then, the candidate model is accepted with a probability which is computed as the ratio of the likelihood of the candidate and current models. Such approach has advantages and drawbacks. In this way, the proposal and prior distributions cancel out in the acceptance term (equation (7)), and they do not need to be explicated. The main advantage is that this recipe can be used in case of complicated and correlated prior and proposal distributions. On the other hand, the main drawback is related to the reduced efficiency of this sampling strategy in case where the posteriors are strongly different from the priors, which means that the data contain a large amount of information on the model parameters. We observe that, in case where the sensitivity of one data set on the overall structure is lower that the other, limited information is anticipated, at least for one of the two observables, thus resulting in a wide posterior not that different from the prior for the poorly resolved parameters. In such cases, the Mosegaard and Tarantola (1995) approach does not suffer from limited efficiency as demonstrated by the acceptance ratio along the chains that is as high as 0.37 in both our test cases.

For sake of completeness, we discuss now the possibility of using the SD algorithm in the case where a single observable is used to constrain two different physical parameters. For example, this is sometimes the case for single RF inversion, where a 1‐D *S* wave velocity profile is usually defined together with a 1‐D *V*_*P*_/*V*_*S*_ ratio profile (e.g., Di Bona et al., 2008; Piana Agostinetti & Malinverno, 2018). In general, a single observable has different sensitivity to variations in different parameters, where often a first parameter is more robustly resolved and a second one is only partially constrained from the field measurements (e.g., in the case of RF, *V*_*P*_/*V*_*S*_ ratio is generally considered less constrained than *V*_*S*_ velocity; see Licciardi et al., 2014.) In this case, the SD algorithm can be adopted to decouple the structure of the two physical properties even in the case of one single data set and to point out the different sensitivity. As mentioned above, the inversion of a RF data set could be able to constrain only an average crustal *V*_*P*_/*V*_*S*_ ratio, while crustal layering could be defined by the *V*_*S*_ profile. This fact does not mean that all crustal layers have the same *V*_*P*_/*V*_*S*_ ratio, which is definitely unrealistic, but it should give an insight on the different resolution on the two physical properties.

In this study, we presented our algorithm in the 1‐D case. However, this algorithm is general and can be easily extended to the 2‐D or 3‐D cases. The RjMcMC algorithms have been used to recover 2‐D and 3‐D fields (e.g., Bodin, Sambridge, Tkalcic, et al., 2012; Galetti & Curtis, 2018; Piana Agostinetti et al., 2015). In this case, the field to be reconstructed is parameterized in terms of a variable number of Voronoi cells that are defined by a set of Voronoi nodes which can be moved within the investigated space. For a joint inversion of two observables and two properties in 2‐D/3‐D using a SD algorithm, we will have three different sets of Voronoi nodes, each associated with a different quality (*Q*_*i*_,*i* = 1,2,3, where, following the notation adopted for the 1‐D problem, *Q*_1_ Voronoi nodes are associated to both properties, and *Q*_2_ and *Q*_3_ Voronoi nodes are associated each to a single property). To reconstruct the 2‐D map or 3‐D volume of a given property, two of the three sets of Voronoi nodes, *Q*_*j*_,*j* = 1,2 or *Q*_*k*_,*k* = 1,3, are merged to define a unique Voronoi tessellation. The recipe adopted here can be kept without any modification, but some changes could be needed to increase the efficiency, for example, moves 12 and 13 should consider only neighboring Voronoi cells (see Appendix A). Exploring a 2‐D/3‐D space would probably make worse the key issue for the SD algorithm: computation time. In Bodin, Sambridge, Rawlinson, et al. (2012), an alternative strategy is proposed for the specific geophysical inversion, to speed‐up computation in standard RjMcMC algorithm applied to a 2‐D inverse problem. Similar solutions should be seek for an efficient SD algorithm in 2‐D/3‐D.

Other geophysical applications will be tested like Local Earthquake Tomography, where *P* and *S* waves first arrival are used to constrain local 3‐D *P* and *S* waves velocity models. Due to the larger uncertainties associated to *S* wave first arrival measures with respect to *P* wave first arrival measures and to the combined effects of fluids, cracks, and lithology on *S* wave velocity, a strong degree of decoupling is anticipated between the *P* and *S* wave velocity models. A 3‐D development of the SD algorithm will be able to focus on this geophysical inverse problem to find out coupled and decoupled regions in the investigated rock volume.

## 5 Conclusions

We develop an algorithm (called SD algorithm) for a probabilistic evaluation of the spatial coupling between *N*_prop_ different physical parameters within the same volume, from the inversion of an arbitrary number of data sets. Our algorithm, based on a RjMcMC approach, produces Bayesian inference about the investigated properties and, potentially, their common distribution in space/time, if similar structures are supported by the data. If the data do not support the same spatial/temporal distribution, the algorithm introduces separated structure for each different physical quantity.

The algorithm has been tested in the case of two different 1‐D geophysical inverse problems. Our main findings are

- Our SD algorithm outperforms classical McMC algorithm in recognizing portions of the investigated 1‐D interval where two different properties are not sharing the same structure.
- In case of fully coupled structures, our algorithm produces comparable solutions to those offered by standard approaches.
- The algorithm has been positively tested on both a standard “changepoints” inverse problem with optimal data coverage and a more complex geophysical joint inversion with limited data.

## Appendix A Candidate Model Selection

### A.1

In this study, developed for *N*_prop_=2, our recipe is composed of 19 moves, where each move perturbs the model in some of its parameters. During the McMC sampling, one move is randomly selected out of the 19 moves, and the current model is perturbed according to such move. Perturbing the current model using only one move at a time guarantees that the perturbation is kept to a “weak” level and allows to exactly monitor the acceptance rate of each move.

Move block 1.: Weak moves ‐ Move 1: Perturb the first *π*_*α*_ hyper‐parameter, which scales the errors on the first observed data set. Here we adopt the approach described in Bodin, Sambridge, Tkalcic, et al. (2012) where the Covariance matrix of the error is kept fixed throughout the McMC and it is scaled using the hyper‐parameter. (Here $\dim(m_{cur})=\dim(m_{cand})$, and the probability for this move is equal to 0.04.) ‐ Move 2: Perturb the second *π*_*β*_ hyper‐parameter which scales the errors on the second observed data set ( $\dim(m_{cur})=\dim(m_{cand})$, 0.04). ‐ Move 3: Perturb the parameter which represents first physical property in the half‐space ( $\dim(m_{cur})=\dim(m_{cand})$, 0.01). ‐ Move 4: Perturb the parameter which represents second physical property in the half‐space ( $\dim(m_{cur})=\dim(m_{cand})$, 0.01). ‐ Move 5: Perturb the depth position of one interface. The depth of one interface is slightly perturbed according to the uniform prior distribution, following the approach described in Piana Agostinetti and Malinverno (2010). The interface cannot be moved closer to another interface than a minimum distance, given by the highest expected resolution of the two observables. While this minimum distance is not a general requirement for a trans‐D sampler (see Bodin & Sambridge, 2009, and Figure S1), its main effect is to drastically reduce the computation time. It is worth mentioning that the symmetry of the proposal is not violated with this minimum distance constraint ( $\dim(m_{cur})=\dim(m_{cand})$, 0.10). ‐ Move 6: Perturb the profile for the first physical property. All the interfaces in classes *Q*_1_ and *Q*_2_ have their first physical property perturbed ( $\dim(m_{cur})=\dim(m_{cand})$, 0.10). ‐ Move 7: Perturb the profile for the second physical property. All the interfaces in class *Q*_1_ have their second physical property perturbed, and all the interfaces in class *Q*_3_ have their first physical property perturbed ( $\dim(m_{c]ur})=\dim(m_{cand})$, 0.10).
Move block 2.: Intermediate moves ‐ Move 8: Birth of an interface for parameter *α* at a location where there is already an interface in *β*. In this case, any additional structure is added to the general model, and an interface in class *Q*_3_ simply becomes an interface in class *Q*_1_ ( $\dim(m_{cand})=\dim(m_{cur})+1$, 0.05). ‐ Move 9: Death of an interface for parameter *α* at a location where there are both interfaces in *α* and *β*. Similar to move 8, an interface in class *Q*_1_ becomes an interface in class *Q*_3_ ( $\dim(m_{cand})=\dim(m_{cur})-1$, 0.05). ‐ Move 10: Birth of an interface for parameter *β* at a location where there is already an interface in *α*. In this case, any additional structure is added to the general model and an interface in class *Q*_2_ simply becomes an interface in class *Q*_1_ ( $\dim(m_{cand})=\dim(m_{cur})+1$, 0.05). ‐ Move 11: Death of an interface for parameter *β* at a location where there are both interfaces in *α* and *β*. Similar to move 10, an interface in class *Q*_1_ becomes an interface in class *Q*_2_ ( $\dim(m_{cand})=\dim(m_{cur})-1$, 0.05). ‐ Move 12: Merging two single‐property interfaces. In this case, one randomly picked interface in class *Q*_2_ and one randomly picked interface in class *Q*_3_ are joined to form an interface in class *Q*_1_. Note that the two interfaces do not need to be close to each other. The depth of the new interface in class *Q*_1_ is set equal to the depth of one of the two old interfaces ( $\dim(m_{cand})=\dim(m_{cur})-1$, 0.05). ‐ Move 13: Splitting an interface in class *Q*_1_. In this case, one interface in class *Q*_1_ is splitted in two interfaces, one in class *Q*_2_ and one in class *Q*_3_. One of the two new interfaces keeps the depth of the old interface in class *Q*_1_, while the other one is randomly placed within the prior distribution ( $\dim(m_{cand})=\dim(m_{cur})+1$, 0.05).
Move block 3.: Strong moves ‐ Move 14: Birth of a new interface in class *Q*_1_. A new interface is placed at a randomly generated depth. Its physical properties are randomly extracted from the correspondent prior probability distribution ( $\dim(m_{\mathrm{cand}})=\dim(m_{cur})+3$, 0.05). ‐ Move 15: Remove one interface in class *Q*_1_. One randomly selected interface is removed from class *Q*_1_. No adjustment to the physical properties is given ( $\dim(m_{cand})=\dim(m_{cur})-3$, 0.05). ‐ Move 16: Birth of a new interface in class *Q*_2_. As for move 8, but an interface in class *Q*_2_ is added ( $\dim(m_{cand})=\dim(m_{cur})+2$, 0.05). ‐ Move 17: Remove one interface in class *Q*_2_. As for move 9, but an interface in class *Q*_2_ is removed ( $\dim(m_{cand})=\dim(m_{cur})-2$, 0.05). ‐ Move 18: Birth of a new interface in class *Q*_3_. As for move 8, but an interface in class *Q*_3_ is added ( $\dim(m_{cand})=\dim(m_{cur})+2$, 0.05). ‐ Move 19: Remove one interface in class *Q*_3_. As for move 9, but an interface in class *Q*_3_ is removed ( $\dim(m_{cand})=\dim(m_{\mathrm{cur}})-2$, 0.05).

#### A1 A General Recipe for a SD Algorithm With N_prop_ Physical Properties

Our recipe can be generalized for decoupled‐coupled models with *N*_prop_ physical properties. In this case, the number of classes of interfaces is NC=∑i=1NpropiNprop. The number of possible moves is clearly a function of the number of physical properties. Following the scheme adopted above, we can write the three blocks of moves as

(A1) $$N_{1}=1+3\times N_{\mathrm{prop}},$$

(A2) N2=4×∑i=1Nprop−1iNprop(iNprop−i),

(A3) N3=2×∑i=1NpropiNprop,

where *N*_1_ is the number of moves belonging to the first block, where one move perturbs an interface depth, *N*_prop_ moves refers to the perturbation of the half‐space, *N*_prop_ moves are used to perturb the profile of one physical property, and *N*_prop_ moves propose a candidate with a different hyper‐parameter (here we are considering the joint inversion of *N*_prop_ observables for *N*_prop_ physical properties). The second block has *N*_2_ potential moves, where half of them refers to add or remove a parameter to one interface in one class (and, thus, changing the class of the interface), the second half define the “merging” of two interfaces for two classes to form a new interface in another class, and the reverse move. It is worth noticing that in the last case, we consider only to merge/separate interfaces with one parameter, that is, merging/separating only one parameter. The number *N*_3_ is the number of moves belonging to the third block, thus the birth and death of one interface in one of the *N*_*C*_ classes (i.e., so the number of moves is twice the number of classes of interfaces). For reference, in the cases presented above with *N*_prop_=2: *N*_1_=7, *N*_2_=6, and *N*_3_=6. We computed the number of moves for a model with three parameters, *N*_prop_=3: *N*_1_=10, *N*_2_=14, and *N*_3_=36.

It is clear that adopting our recipe can hugely increase the number potential moves. If many moves are given, it can be difficult assessing the efficiency of each single move and, thus, assessing the detailed performance of the algorithm. Along this line, we can hypothesize that global efficiency of the algorithm should be measured looking to the efficiency of the three blocks of moves more than checking the efficiency of the single move, that is, we have to monitor how the algorithm efficiently moves across different parameterizations more than looking to how the algorithm samples a single physical parameter.
